# HomeSTEAD’s physical activity and screen media practices and beliefs survey: Instrument development and integrated conceptual model

**DOI:** 10.1371/journal.pone.0226984

**Published:** 2019-12-31

**Authors:** Amber E. Vaughn, Derek P. Hales, Cody D. Neshteruk, Dianne S. Ward

**Affiliations:** 1 Center for Health Promotion and Disease Prevention, the University of North Carolina at Chapel Hill, Chapel Hill, NC, United States of America; 2 Department of Nutrition, Gillings School of Global Public Health, the University of North Carolina at Chapel Hill, Chapel Hill, NC, United States of America; Middlesex University, UNITED KINGDOM

## Abstract

The home environment has a significant influence on children’s physical activity and obesity risk. Our understanding of this environment is limited by current measurement tools. The Home Self-administered Tool for Environmental assessment of Activity and Diet addresses this gap. This paper describes the development and psychometric testing of its family physical activity and screen media practices and beliefs survey. Methods: Survey development was guided by the Analysis Grid for Environments Linked to Obesity (ANGELO) framework and informed by a literature review, expert opinion, and cognitive interviews. Parents of children ages 3–12 years (n = 129) completed the HomeSTEAD survey three times over 12–18 days. Additionally, parents reported on child behaviors and trained staff measured parent and child height and weight. Five exploratory factor analyses were conducted after categorizing items into: control of physical activity, control of screen media, explicit modeling, implicit modeling, and perceived barriers and facilitators. Scales with 3 or more items underwent scale reduction. Psychometric testing evaluated internal consistency (Chronbach’s alphas), test-retest reliability (analysis of variance and intraclass correlations (ICC)), and construct validity (correlations with child BMI, physical activity, screen time). An integrated conceptual model of parent physical activity and screen media practices and beliefs was developed based on recent literature to aid in the identification and naming of constructs. Results: Final scales demonstrated good internal consistency (median Cronbach’s alpha = 0.81, IQR = 0.74–0.85), test-retest reliability (median ICC = 0.70, IQR = 0.66–0.78), and construct validity (with correlations between scale score and children’s behaviors generally in the expected direction). Comparison with the integrated conceptual model showed that most identified constructs were captured. Conclusions: The family physical activity and screen media practices survey advances the measurement of the home environment related to children’s physical activity, screen time, and weight. The integrated conceptual model provides a useful framework for researchers studying both physical activity and screen media parenting practices.

## Introduction

Nearly a third (31.8%) of children in the United States are overweight or obese [1], and similar rates are observed worldwide [2]. Insufficient physical activity and excess sedentary time, particularly in the form of screen media use, are widespread among children and contribute to their development of obesity [3–7], which in turn lead to numerous health, social, and psychological problems [8–10]. Physical inactivity, screen media use, and obesity generally track from childhood into adolescence and adulthood [11–15]; hence, intervention strategies targeting young children are needed to promote physical activity, reduce sedentary behavior and screen media use, and prevent their development of obesity.

The home environment provides an ideal setting to influence children’s physical activity, sedentary and screen media behaviors and thereby reduce their risk of obesity. Several physical and social factors within the home have been shown to be influential. Aspects of the physical environment such as availability of play equipment is positively associated with children’s physical activity [16–18], while the presence of media equipment is positively associated with children’s sedentary behavior [19]. Studies of homes’ social environment factors demonstrate that parents’ physical activity and screen media parenting practices play an important role in shaping their children’s physical activity and media habits. Modeling of physical activity, providing logistic support for physical activity, and encouraging activity are associated with increased physical activity in children [20–23]. In addition, role modeling responsible screen media behaviors (e.g., limiting own video game use) and enforcing screen media rules are associated with less screen media use [24]. However, co-viewing between parent and child (e.g., without proper parent engagement) and restricting television (TV) are associated with more screen media use [25, 26].

Despite the importance of the home environment in preventing obesity through the promotion of healthy physical activity and screen media behaviors, there are few measures that adequately assess the physical and social environment of the home. A systematic review of measures of the home environment found that existing measures have rarely undergone extensive validity or reliability testing and few offer a comprehensive assessment of the home environment [27]. In the field of physical activity and screen media parenting specifically, psychometrically sound, comprehensive, and theoretically driven measures of physical activity and screen media parenting practices are also lacking [28–31]. There has been only limited development of measures since these systematic reviews [32]. Concerns have also been raised that existing measures do not adequately capture the practices that parents are using (e.g., control, modeling, structure) [33]. There is a clear gap in measurement tools, limiting our ability to assess the home environment as it relates to children’s physical activity, screen media and obesity risk.

The Home Self-administered Tool for Environmental assessment of Activity and Diet (HomeSTEAD) was created to address this gap and comprehensively assess the environmental qualities of the home related to children’s physical activity/screen media and diet [34]. Development of the HomeSTEAD tool was guided by the Analysis Grid for Environments Linked to Obesity (ANGELO) Framework, which recognizes four spheres of influence that impact children’s weight and related behaviors: physical, sociocultural, economic, and political [35]. When applying this framework to the home environment, the physical and sociocultural spheres were considered to be most relevant. Thus, the HomeSTEAD tool was designed with four parts: (1) a physical activity and media equipment inventory and (2) a family physical activity and screen media practices and beliefs survey, which together assess the physical and sociocultural environment related to children’s physical activity and screen media; and (3) a food inventory and (4) a family food practices surveys, which assess the physical and sociocultural environment related to children’s diet. The development and psychometric testing of HomeSTEAD’s physical activity and media equipment inventory and family food practices survey have been described elsewhere [34, 36]. This paper describes the development of the HomeSTEAD physical activity and screen media practices and beliefs survey and presents the results of the reliability and validity testing. Specifically, the aims of this paper are to describe item development and efforts to establish face validity and item comprehension, to evaluate how individual items come together into scales, and to examine test-retest reliability and construct validity of those scales. In addition, this paper reviews current conceptual models for physical activity and screen media practices [29, 37, 38], proposes an integrated model, and identifies alignment between constructs from that integrated model and the scales from HomeSTEAD’s physical activity and screen media practices and beliefs survey.

## Materials and methods

HomeSTEAD’s development has been previously described in detail elsewhere [34]; therefore, only the methods most relevant to the development of the family physical activity and screen media practices and beliefs survey are provided. Survey development and psychometric testing were guided by work by DeVellis on scale development s [39]. Reporting on these procedures is guided by the more recently developed COSMIN Study Design Checklist for Patient-Reported Outcome Measurement Instruments [40]. All protocols were reviewed and approved by the University of North Carolina at Chapel Hill Institutional Review Board.

### HomeSTEAD instrument development

Development of the HomeSTEAD survey used a mixed methods approach. As noted above, the ANGELO framework was used to develop a preliminary content map identifying constructs within the home’s sociocultural environment that might influence children’s physical activity and screen media behaviors. The constructs identified as most relevant were parents’ intentional and unintentional behaviors (i.e., practices) as well as their beliefs that influence 3-12-year-old children’s physical activity and screen media behaviors. Then, a systematic review was conducted (in 2009) to refine the content map, identify specific practices and beliefs, and explore existing measures. Existing measures were compiled into a database which was labeled and organized using the constructs identified in the content map. When there was overlap in existing items, two members of the research team reviewed available items and selected the items they agreed were most relevant for that construct. When existing items were not available, the research team drafted new items. When possible, response options were standardized. For example, physical activity and screen media items generally used 6-point Likert-type response scales (i.e., 1 = never, 2 = rarely, 3 = occasionally, 4 = sometimes, 5 = often, 6 = very often; or 1 = strongly disagree, 2 = disagree, 3 = slightly disagree, 4 = slightly agree, 5 = agree, 6 = strongly agree).

Two scientific experts in parent physical activity and screen media practices and beliefs research reviewed the initial set of items to assess face validity (March-April 2010). These experts reviewed the draft instrument individually, using a word document, and were asked to add feedback and suggestions related to content coverage, item relevance and intention, and question format and clarity. The survey was refined based on their feedback.

One-on-one cognitive interviews were conducted with parents of 3-12-year-old children (April-August 2010). A convenience sample of parents was recruited through newspaper advertisements, listserv notifications, and community postings. To be eligible, parents had to have at least one child 3–12 years old with no physical/heath limitations affecting their diet or physical activity, live within 30 miles of the research campus, and be able to speak English. To minimize participant burden, each interview focused on one of the four parts of the HomeSTEAD tool. Parents were guided through the survey by interviewers trained specifically for this study, who prompted parents to provide feedback on item clarity and comprehension. Interviews were not recorded, but the structured interview guide allowed interviewers to easily make note of any problematic items and confusion about question intention. After completing six interviews with the family physical activity and screen time practices survey, a summary report was prepared and problematic items were reviewed and revised by the research team. An additional round of cognitive interviews was conducted with five parents to ensure the revised items were acceptable and no additional revisions were needed. The preliminary HomeSTEAD tool included 240 items assessing the physical activity and screen media parenting practices and beliefs.

### Reliability and validity testing

Reliability and validity testing were conducted with a convenience sample of 129 families (October 2010 –May 2011). A convenience sample of families was recruited through newspaper advertisements, listserv notifications, and community postings. To be eligible, families needed at least one child between the ages of 3–12 years without physical or health limitations, live within 30 miles of the research project office, and have a parent able to speak English. For families with more than one child within the target age range, one child was chosen by the research team to be the reference child, often the older child, to ensure equal distribution of child ages.

Participants completed all four parts of the HomeSTEAD tool at three different time points over the course of 12 to 18 days and allowed research staff to complete an in-home observation. Participants were mailed the Time 1 HomeSTEAD survey along with a demographic survey, child physical activity screener, and consent form two to three days before the home observation. The child physical activity screener asked parents to report on the time their child spend in various activities (i.e., playing outside, watching TV, playing video games) on weekdays and weekend days during a typical week. At the home visit, two trained staff members collected the Time 1 surveys and completed the home observation. The home observation was designed to assess the physical environment (e.g., play equipment, media devices). It was not possible to directly observe parenting practices and beliefs due to the limited opportunity to assess typical practices during a relatively short home observation and the inability to observe parent beliefs. The reference child’s height was measured to the nearest 1/8 inch using a Shorr or Seca stadiometer (Shorr Productions, Olney, MD; Seca Corporation, Columbia, MD) and weight was measured to the nearest 0.1 pound using a Seca portable electronic scale (model 770 or 874, Seca Corporation, Columbia, MD). Height and weight data were later used to calculate child BMI and BMI percentile using Centers for Disease Control and Prevention growth charts [41]. At the conclusion of the home observation, research staff distributed the Time 2 HomeSTEAD survey, instructing participants to return the survey via mail within 24 hours. Approximately 10 days later, the Time 3 survey was mailed to participants with instructions to complete and return the survey within four days. If the Time 3 survey was not completed and returned within an additional 10 days (even after reminder phone calls), that participant’s data were not included in the analysis.

### Statistical analysis

Identifying, refining, and evaluating the potential scales contained within the survey involved a process of item assessment, exploratory factor analysis, scale reduction, and examination of external relationships of interest. Initial analyses were conducted in 2014, then revisited and refined in 2017–2018. First, items were examined to assess missingness, response variability, and relationships with other items. Items were flagged if >80% of responses fell within two response categories or if >75% of responses fell within one response category, indicating low variability. Items were also flagged if the correlation with other items was 0.75 or higher, indicating high similarity between items.

Because development of the survey was based on a reflective model, including multiple items about the same underlying construct, Exploratory Factor Analyses (EFA) were used to examine how items contributed to factors assessing different physical activity and screen media parenting practices and beliefs. Given the large number of items (n = 240) and limited sample size (n = 129), testing a single EFA model was not possible. Based on our earlier work selecting and developing items, two strategies were identified for pre-sorting items: (1) to categorize items as physical activity practices and beliefs or screen media practices and beliefs, and (2) to categorize items as specific types of parenting practices noted in the literature (e.g., control, explicit modeling, implicit modeling, perceived barriers). Preliminary EFA analyses examined both strategies. While there was overlap in the factors that emerged, the latter approach was preferred as it yielded a clearer differentiation of relevant constructs based on the growing literature in this area. It also allowed for larger participant to item ratios, potentially resulting in more stable factors. Hence, five EFAs were conducted after pre-sorting item into the following categories: control of physical activity, control of screen media, explicit modeling, implicit modeling, and perceived barriers and facilitators. Control of physical activity and screen media included items where parents exert control over children’s behavior (e.g., rules/restrictions, rewarding). Implicit modeling items examined specific attitudes (e.g., importance/value of activity), while explicit modeling items included parent behaviors (e.g., verbal encouragement, prompting, modeling activity). Perceived barriers and facilitators assessed interpersonal (e.g. child preference for activity) and intrapersonal (e.g. influence of other adults) factors that may influence children’s physical activity and media use.

Factor solutions were evaluated based on eigenvalues, scree plots, and interpretability criteria (e.g., comparative fit index, root mean square of approximation) [42, 43]. Items with low factor loadings for all identified factors, or larger cross loadings, were eliminated one or two at a time (items with lowest factor loading eliminated first). The EFA was then repeated in this iterative process until all items loaded substantially (>0.35) on at least one factor. During this process, items that had been previous flagged for low variability or high correlations received extra scrutiny. If an item cross-loaded (>0.40 on multiple factors), it was included in the factor with the higher loading.

Given the need for parsimony in self-administered surveys, scales with three or more items were examined for possible item reduction [42, 44]. Multiple reduced versions of the scale were examined. First, a best subset regression model was used to predict the original scale score from the individual items. This allowed us to identify the most “important” items in the original scale score, the fewest number of items needed to best represent the original scale score, and the interchangeability of items within the reduced scale. Additional criteria considered included the factor loadings from the original EFA (giving preference to items with higher loadings), the internal consistency of the reduced scale compared to the original (giving preference to reduced scales with Cronbach’s alpha >0.7) [45], and the correlations of original and reduced scales with external criteria (e.g., child BMI, physical activity, screen time; giving preference to reduced scales with larger correlations, suggesting greater construct validity). Hypotheses for this construct validity component were that controlling practices and perceived barrier beliefs would be negatively associated with the target behavior (e.g., higher use of control of physical activity by parents would be associated with lower child physical activity), while explicit and implicit modeling and perceived facilitator beliefs would be positively associated with the target behavior.

Scale scores were then calculated by averaging the individual items (i.e., Likert responses) within each factor, resulting in a continuous score for each scale. Scores were computed even if some component items were missing. Overall, an average of 6% of items responses were missing. Most of the missing responses were associated with questions about video game use, which parents of young children did not see as applicable (accounted for 24% of all missing data). Due to missing item level data, 2% of scores could not be computed. Again, this primarily affected scores related to computer and video game practices and beliefs. Scores were computed for each time point (Time 1, 2 and 3). For all scales, higher scores reflect greater use of a practice or greater agreement with a belief. Mean differences over time were tested using repeated measures analysis of variance (ANOVA); single-measure intraclass correlations (ICC) were calculated to examine test-retest reliability. The single-measure ICC, ICC(1,1) from Shrout and Fleiss [46], estimates reliability given a single random administration. ICCs of 0.61–0.80 indicate moderate agreement, and ICCs of 0.81–1.00 indicate substantial agreement [47]. The EFAs were conducted using Mplus, versions 7 and 8 (Muthén & Muthén, Los Angeles, CA). All other cleaning and analyses were done using SAS^®^ software, version 9.4 (SAS Institute, Cary, NC).

### Integrated conceptual model development

To inform the naming of final scales and ensure consistency with the broader literature, it was necessary to develop an integrated conceptual model of physical activity and screen media parenting. Development of this integrated model was based heavily on conceptual models presented in three recently published papers, including two models on physical activity parenting and one on screen media parenting [29, 37, 38]. These papers represent collaborative efforts between leading researchers from multiple institutions to identify and define constructs related to physical activity or screen media parenting. Since existing conceptual models address either physical activity or screen media parenting, authors created an integrated conceptual model that clearly identified both physical activity and screen media practices and beliefs and proposed terminology that facilitated alignment of similar parenting constructs across physical activity and screen media parenting.

## Results

### Sample descriptives

Parents in the study sample (n = 129) were predominately mothers (91%) and represented a mix of racial and income groups. The majority was white (71%) or African American (25%), had a household income above the area’s median (68% with annual household income ≥$50,000), and were well-educated (79% college degree or higher). Children in the sample included similar numbers of boys and girls (51% vs 49%, respectively), who were on average 7.1 ±2.9 years old, and had a BMI percentile of 59.6 ±27.1. Compliance with study protocols was high with 125 parents (97%) completing all three self-administrations of the survey and the home observation. Participants also completed the surveys in a timely manner matching the intended time interval between administrations. On average, there were 3.9 ±3.7 days between Time 1 and Time 2 surveys and 12.4 ±5.6 days between Time 2 and Time 3.

### Factor analysis and internal reliability

The initial set of five EFAs identified 32 factors and retained 196 of the 240 items, including four factors (31 items) related to control of physical activity, six factors (45 items) related to control of screen media, seven factors (46 items) related to explicit modeling, seven factors (38 items) related to implicit modeling, and eight factors (36 items) related to perceived barriers and facilitators. These original factors and items are provided in S1 Table. Twenty four of the 32 factors had greater than three items per factor and were examined for scale reduction, during which 42% of items were eliminated. Specifically, control of physical activity scales were reduced from 31 to 14 items; control of screen media scales were narrowed from 45 to 26 items; explicit modeling scales were reduced from 46 to 24 items; implicit modeling scales were trimmed from 38 to 27 items; and perceived facilitators and barriers scales were narrowed 36 to 23 items; resulting in a final instrument with 114 items and 32 scales. Tables 1 and 2 provide the items, factor loadings, Cronbach’s alphas, and mean and standard deviation of scores (Time 1 data) for these final scales. Correlations between final scales are provided in S2 Table.

**Table 1:** Factor loadings for final reduced scales, internal consistency (Cronbach’s alpha), mean (SD) scores (Time 1)–Control of PA and Screen Media scales.

Scale Name and Items	Factor loading^1^	Internal consistency	T1 score mean (SD)	T1, T2, T3 ICC (mean score/ single score)
**Control of Physical Activity**				
***Weather-related restriction of outdoor play***		0.90	3.51 (1.35)	0.93/0.82
How often do you allow your child to play outside if it is wet? (R)	0.90			
How often do you allow your child to play outside if it is raining? (R)	0.83			
How often do you allow your child to play outside on cold days? (R)	0.78			
***Restriction of active play indoors***		0.81	2.03 (0.54)	0.89/0.73
When are these activities allowed when your child is playing inside?^2^				
Running around	0.87			
Chasing	0.79			
Piling up pillows and jumping on them	0.62			
Jumping from a height	0.61			
***Use of physical activity as a bribe***		0.70	1.89 (0.88)	0.87/0.69
How often do you take outside time away from your child for bad behavior?	0.73			
How often do you use sports or physical activities to control your child’s behavior?	0.64			
How often do you use sports or physical activities to get your child to do something?	0.64			
How often so you use physical activity as a punishment for bad behavior?	0.39			
***Perceived influence on physical activity***		0.69	5.17 (0.72)	0.84/0.64
I have influence over how much physical activity my child gets.	0.70			
I have influence over how much my child plays outside.	0.69			
I have little control over how much physical activity my child gets. (R)	0.48			
**Control of Screen Media**				
***Limits on and supervision of screen media***		0.86	4.19 (1.24)	0.94/0.85
Do you limit the amount of time your child watches TV or videos during ***weekend***? If yes, how much? (R)	0.80			
Do you limit the amount of time your child watches TV or videos during ***week***? If yes, how much? (R)	0.65			
Do you limit the amount of time your child uses the computer during ***weekend***? If yes, how much? (R)	0.71			
Do you limit the amount of time your child uses the computer during ***week***? If yes, how much? (R)	0.72			
Do you limit the amount of time your child plays video games during ***weekend***? If yes, how much? (R)	0.62			
Do you limit the amount of time your child plays video games during ***week***? If yes, how much? (R)	0.51			
My child is allowed to turn on the computer without permission. (R)	0.59			
How often is your child supervised when playing video games?	0.45			
How often is your child supervised when watching TV?	0.41			
***Monitoring and use of TV as a threat or bribe***		0.80	3.54 (1.17)	0.91/0.77
How often do you use TV time to get your child to do something?	0.68			
How often do you take away TV, video, or movie time as a punishment for bad behavior?	0.53			
If I do not ***regulate or guide*** my child’s TV watching, s/he would watch too much.	0.52			
If I did not ***monitor*** my child’s TV viewing, s/he would watch too much TV?	0.52			
***Monitoring and use of video games as a threat or bribe***		0.87	2.76 (1.39)	0.91/0.77
If I do not ***regulate or guide*** my child’s video game play, s/he would play too much.	0.87			
If I did not ***monitor*** my child’s video game play, s/he would play too much?	0.84			
How often do you use video game time to get your child to do something?	0.74			
How often do you use video games to control your child’s behavior?	0.67			
***Use of computers as a threat or bribe***		0.81	2.33 (1.36)	0.85/0.65
How often so you use computer time to get your child to do something?	0.94			
How often do you take away computer time as a punishment for bad behavior?	0.81			
How often do you offer video game time (handheld or console) as a reward for good behavior?	0.41			
***Negotiation of screen media rules***		0.84	0.67 (0.41)	0.93/0.81
Who is responsible for enforcing rules related to TV viewing, video game playing, or computer use?	0.96			
Who is responsible for setting rules related to TV viewing, video game playing, or computer use?	0.93			
Who is responsible for deciding when your child can watch TV, play video games, or use the computer?	0.92			
***Perceived influence on screen media use***		0.84	5.55 (0.64)	0.83/0.62
I have influence over how much my child watches TV, plays video games, and uses the computer.	0.91			
I have influence over how much my child plays video games.	0.70			
I have influence over how much television my child watches.	0.62			

^1^ Factor loadings reported are from the original scales (not reduced)

^2^ Response options were 1 = anytime, 2 = sometimes, 3 = never

(R) indicates items are reverse coded

**Table 2:** Factor loadings for final reduced scales, internal consistency (Cronbach’s alpha), mean (SD) scores (Time 1)–Explicit and Implicit Modeling and Perceived Barriers and Facilitators scales.

Scale Name and Items	Factor loading^1^	Internal consistency	T1 score mean (SD)	T1, T2, T3 ICC (mean score/ single score)
**Explicit Modeling**				
***Co-participation in physical activity***		0.75	3.47 (1.19)	0.91/0.78
How often does your family play outdoors as a form of family recreation?	0.68			
How often do you or another adult in the household start a physically activity game with your child?	0.66			
How often does your family use sport/physical activity as a form of family recreation?	0.51			
***Encouragement for outside play***		0.74	4.34 (1.01)	0.87/0.69
During a typical week, how often do you or another adult in the household encourage your child to play outside?	1.0			
During a typical week, how often do you or another adult in the household try to get your child to play outside when the weather is nice?	0.61			
How often do you or another adult in the household send your child outside to play?	0.49			
***Facilitation of sports and lessons***		0.74	2.40 (1.36)	0.86/0.67
How often in the past 7 days did you or another adult in the household watch your child’s sporting events, lessons, or other organized physical activities with them?	0.81			
How often in the past 7 days did you or another adult in the household take your child to practice, lessons, classes, or other programs that involved moderate or vigorous physical activity?	0.78			
During the past year, has an adult in your household enrolled your child in lessons, classes, or sports involving moderate or vigorous physical activity?	0.44			
***Encouragement and education to reduce screen media***		0.83	3.88 (1.21)	0.86/0.66
How often do you or another adult in the household discuss with your child how sedentary habits can be unhealthy?	0.76			
During a typical week, how often do you or another adult in the household say things to encourage your child to spend less time being sedentary?	0.75			
How often do you or another adult in the household discuss with your child how watching too much TV can be unhealthy?	0.70			
***Co-viewing TV***		0.75	3.10 (0.99)	0.93/0.81
How often do you or another adult in the household watch TV with your child?	0.71			
How often does your family watch TV or movies as a form of family recreation?	0.63			
How often does your child see you or another adult in the household watching TV/movies?	0.47			
During a typical week, how often do you or another adult in the household ask your child to watch TV with you?	0.44			
***Co-use of video games and computer***		0.69	2.13 (0.92)	0.91/0.77
How often do you or another adult in your household play video games with your child?	0.82			
How often does your family play video games as a form of family recreation?	0.74			
How often do you or another adult in your household use the computer with your child?	0.47			
***Context driven permissiveness for screen media***		0.85	3.02 (1.12)	0.92/0.79
How often do you or another adult in the household turn on the TV, video, or a movie for your child so you can get things done around the house?	0.77			
When my child watches TV, it helps me get things done around the house.	0.74			
When my child uses the computer, it helps me get things done around the house.	0.68			
When my child plays video games, it helps me get things done around the house.	0.59			
When my child is bored, it helps to turn on a video game.	0.52			
**Implicit Modeling**				
***Value of parent physical activity***		0.88	5.12 (0.87)	0.91/0.78
Participating in physical activity is valuable to me.	0.90			
Participating in regular physical activity is important to me.	0.81			
I look forward to being physically active.	0.74			
I do not enjoy being physically active in my free time.(R)	0.71			
***Value of child sports***		0.74	4.53 (1.09)	0.89/0.74
My child benefits from playing sports.	0.91			
How important is it that your child participates in organized sports and activities?	0.59			
***Value of child physical activity***		0.77	5.44 (0.60)	0.86/0.68
Children who do regular physical activity are more healthy.	0.77			
My child benefits from being physically active.	0.74			
How important is it that your child does physical activities in his/her free time?	0.43			
***Health benefits of child physical activity***		0.82	5.33 (0.68)	0.80/0.57
Children who do regular physical activity are less stressed.	0.87			
Children who do regular physical activity are less likely to be overweight.	0.81			
Children who do regular physical activity are happier.	0.59			
***Value of TV for parent***		0.82	3.16 (1.17)	0.91/0.77
Watching TV is important to me.	0.91			
Watching TV is valuable to me.	0.83			
Watching TV is good entertainment for my child.	0.52			
***Value of child screen media***		0.65	2.30 (0.83)	0.87/0.69
How important is it that your child be able to play video games during their free time?	0.72			
How important is it that your child be able to watch TV or movies during their free time?	0.67			
I enjoy playing video games with my child.	0.53			
My child benefits from using the internet.	0.47			
***Entertainment and education benefits of child screen media***		0.76	3.52 (0.90)	0.87/0.68
Using the computer is good entertainment for my child.	0.80			
Playing video games is good entertainment for my child.	0.75			
Playing video games helps my child learn.	0.65			
Watching TV helps my child learn.	0.36			
**Perceived Barriers and Facilitators**				
***Child preference for inactivity***		0.61	2.19 (0.74)	0.85/0.66
When outside, my child prefers… (light play ↔ very active play)	0.60			
My child’s physical activity is limited due to my child’s lack of interest or motivation.	0.51			
What does your child usually do when s/he has a choice about how to spend his/her free time?	0.51			
***Lack of support for physical activity from adults***		0.60	2.41 (0.96)	0.83/0.61
My child’s physical activity is limited due to lack of adult supervision.	0.65			
My child’s physical activity is limited due to my own lack of motivation and interest.	0.47			
My child’s physical activity is limited due to other adults in my child’s life.	0.44			
***Lack of self-efficacy for limiting screen media***		0.85	2.08 (0.95)	0.86/0.67
It is hard to limit the amount of time my child spends on the computer.	0.88			
When I am tired it is hard to get my child to watch less TV.	0.81			
It is hard to limit the amount of video games my child plays.	0.74			
My child’s begging or nagging makes it difficult to get him/her to play video games less.	0.71			
It is hard to limit the amount of TV my child watches.	0.71			
***Permissiveness for TV viewing by other adults***		0.87	2.38 (1.31)	0.90/0.75
Other adults in my child’s life make it difficult to enforce household rules about TV viewing.	0.69			
Other adults in my child’s life make it difficult to get my child to watch less TV.	0.68			
***Permissiveness for screen media by other adults***		0.87	1.92 (1.08)	0.88/0.70
Other adults in my child’s life make it difficult to enforce household rules about computer use.	0.89			
Other adults in my child’s life make it difficult to get my child to play on the computer less.	0.85			
Other adults in my child’s life make it difficult to get my child to play video games less.	0.44			
***Enforcement of screen media rules by other adults***		0.76	3.74 (1.48)	0.90/0.76
Other adults in my household tightly enforce the household rules related to video game play/use.	0.70			
Other adults in my household tightly enforce the household rules related to computer use.	0.62			
Other adults in my household tightly enforce the household rules related to TV viewing.	0.50			
***Weather-related barriers to physical activity***		0.82	3.18 (1.04)	0.77/0.53
My child’s physical activity is limited due to cold weather.	0.94			
My child’s physical activity is limited due to hot weather.	0.71			
***Family consistency in beliefs about screen media***		0.92	4.80 (1.15)	0.80/0.57
You or another adult in the household have the same views about computer use.	0.97			
You or another adult in the household have the same views about video game playing.	0.81			
You or another adult in the household have the same views about how much TV child should watch	0.79			

^1^ Factor loadings reported are from the original scales (not reduced)

(R) indicates items are reverse coded

#### Control of physical activity scales

Control of physical activity scales included weather-related restriction of outdoor play, restriction of active play indoors, use of physical activity as a bribe, and perceived influence on physical activity. Final reduced scales had either three or four items and acceptable internal consistency (Cronbach’s α = 0.69 to 0.90). One item from the factor use of physical activity as a bribe had a factor loading slightly below 0.4 that was retained as it was conceptually consistent with the construct being measured.

#### Control of screen media scales

Control of screen media scales included limits on and supervision of screen media, monitoring and use of TV as a threat or bribe, monitoring and use of video games as a threat or bribe, use of computers as a threat or bribe, negotiation of screen media rules, and perceived influence on screen media use. Final reduced scales had between three and nine items and acceptable internal consistency (Cronbach’s α = 0.80 to 0.87). When scales were reduced, some cross-loadings did arise between two closely-related factors–monitoring and use of TV as a threat or bribe and monitoring and use of video games as a threat or bribe–suggesting that these could potentially merge into a single factor (correlation = 0.59).

#### Explicit modeling scales

Even though the explicit modeling EFA included items about physical activity and screen time practices, these tended to naturally separate into different factors. Final scales included co-participation in physical activity, encouragement for outside play, facilitation of sports and lessons, encouragement and education to reduce screen media, co-viewing TV, co-use of video games and computer, and context driven permissiveness for screen media. Final reduced scales had between three and five items and acceptable internal consistency (Cronbach’s α = 0.69 to 0.85). Two items–one in the factor for facilitation of sports and lessons and another in co-use of video games and computer–had factor loadings drop slightly below 0.4 in the reduced models; however, both items were retained as they were conceptually consistent with the constructs being measured.

#### Implicit modeling scales

Similar to explicit modeling, the factors that emerged from the implicit modeling EFA seem to naturally separate into physical activity or screen time practices and beliefs. Final scales included value of parent physical activity, value of child sports, value of child physical activity, health benefits of child physical activity, value of TV for parent, value of child screen media, entertainment and education benefits of child screen media. Final reduced scales had between two and four items and acceptable internal consistency (Cronbach’s α = 0.65 to 0.88). One item from the factor entertainment and education benefits of child screen media had a factor loading slightly below 0.4 that was retained as it was conceptually consistent with the construct being measured.

#### Perceived barriers and facilitators scales

Once again, factors that emerged from the perceived barriers and facilitators EFA seem naturally differentiated between physical activity and screen time practices and beliefs. Final scales included child preference for inactivity, lack of support for physical activity from adults, lack of self-efficacy for limiting screen media, permissiveness for TV viewing by other adults, permissiveness for screen media by other adults, enforcement of screen media rules by other adults, weather-related barriers to physical activity, and family consistency in beliefs about screen media. Final reduced scales had between two and five items and acceptable internal consistency (Cronbach’s α = 0.60 to 0.92).

### Test-retest reliability

The ICCs for the reduced scale scores shown in Table 1 and Table 2 generally demonstrated moderate to substantial agreement over the three administrations. The ICCs for a mean score from all three administrations were 0.80 or above (indicating substantial agreement) for all but one factors (i.e., weather-related barriers to physical activity, ICC = 0.77). As expected, ICCs for single administration were slightly lower (~20% decrease), but still generally indicated moderate to substantial agreement (ICC = 0.88 to 0.53).

### Construct validity

All correlations between scale scores and children’s outdoor playtime, screen time (i.e., watching TV, playing video games), and BMI percentile scales are shown in Table 3. Not all are statistically significant but, there was greater consistency when the construct score and child behaviors were directly related (i.e., 7 of the 13 physical activity practices and beliefs scales were significantly correlated with child outside time; 12 of the 19 screen media practices and beliefs scales were significantly correlated with child TV or video game time) providing construct validity evidence for the new reduced scales. Control of Physical Activity scales generally suggested that when parents exerted more control, children had less outdoor playtime and more screen time, which was consistent with hypothesized relationships. Weather-related restriction of outdoor play was negatively associated with outside playtime (r = -0.36 to -0.4) and positively associated with TV time (r = 0.14 to 0.26) as well as child BMI percentile (r = 0.15). Similarly, restriction of active play indoors and use of physical activity as a bribe (higher scores indicating more control) were also positively associated with weekend screen time (r = 0.20 to 0.27). Additionally, parental perceptions that they had great influence and control over their child’s physical activity (higher scores indicating higher control) was negatively associated with outdoor playtime and TV time on weekdays (r = -0.24).

**Table 3:** Correlations^1^ between final scales and child behaviors (with T1 data).

	Child BMI percentile	Outside play time WD/WE	TV time WD/WE	Video game time WD/WE
**Control of Physical Activity**				
Weather-related restriction of outdoor play	**0.15**	**-0.36/-0.41**	0.14**/0.26**	-0.05/0.15
Restriction of active play indoors	0.07	-0.14/-0.16	0.02/**0.24**	0.10/**0.22**
Use of physical activity as a bribe	-0.03	**0.22/0.23**	0.15/**0.27**	0.11/**0.20**
Perceived influence on physical activity	-0.07	-**0.24**/-0.13	**-0.24/-0.32**	0.003**/-0.20**
**Control of Screen Media**				
Limits on and supervision of screen media	-0.01	-0.07/0.02	**-0.45/-0.50**	**-0.18/-0.23**
Monitoring and use of TV as a threat or bribe	0.05	-0.18/-0.04	0.10/0.10	-0.04/0.00
Monitoring and use of video games as a threat or bribe	0.028	0.17/0.24	-0.21/0.08	**0.28/0.26**
Use of computers as a threat or bribe	0.06	0.07/0.04	0.07/0.15	0.08/0.10
Negotiation of screen media rules	0.01	-0.13/0.03	0.04/0.08	0.03/0.16
Perceived influence on screen media use	**-0.14**	-0.12/-0.11	**-0.32/-0.35**	-0.13**/-0.26**
**Explicit Modeling**				
Co-participation in physical activity	-0.11	**0.25/0.32**	-0.06**/-0.20**	-0.13/-0.06
Encouragement for outside play	**-0.25**	0.03/**0.27**	-0.07**/-0.22**	0.09/0.03
Facilitation of sports and lessons	-0.09	**0.17/0.33**	-0.22/-0.03	0.09/0.12
Encouragement and education to reduce screen media	0.06	-0.18/0.10	-0.09/-0.09	0.13/0.16
Co-viewing TV	0.09	0.02/-0.03	**0.38/0.44**	0.08/0.16
Co-use of video games and computer	**0.15**	0.08/**0.20**	0.04/0.14	**0.40/0.33**
Context driven permissiveness for screen media	0.10	-0.05/-0.08	0.17/0.13	-0.01/0.10
**Implicit Modeling**				
Value of parent physical activity	**-0.20**	0.06/0.09	-0.07/-0.04	-0.13/0.08
Value of child sports	-0.03	0.00/0.02	0.14/0.34	0.08/0.14
Value of child physical activity	**-0.20**	0.08/0.08	-0.06/-0.04	-0.07/-0.03
Health benefits of child physical activity	-0.05	**0.23/0.27**	0.00/0.06	-0.13/0.02
Value of TV for parent	0.05	0.04/-0.11	**0.37/0.37**	-0.05/0.03
Value of child screen media	0.03	0.17/**0.20**	**0.24/0.39**	**0.31/0.35**
Entertainment and education benefits of child screen media	-0.13	-0.02/0.01	0.18/**0.220**	**0.09//0.03**
**Perceived Barriers and Facilitators**				
Child preference for inactivity	-0.03	-0.06/-0.10	**0.19/0.21**	-0.04/0.02
Lack of support for physical activity from adults	**0.17**	-0.09/-0.19	0.08/0.18	-0.13/-0.03
Lack of self-efficacy for limiting screen media	**0.16**	0.11/0.02	**0.37/0.25**	-0.03/0.06
Permissiveness for TV viewing by other adults	0.10	0.08/0.07	0.15/0.08	0.01/0.01
Permissiveness for screen media by other adults	**0.14**	0.03/-0.02	0.10/0.05	-0.01/0.02
Enforcement of screen media rules by other adults	-0.02	-0.03/0.15	**-0.35/-0.38**	0.18/0.01
Weather-related barriers to physical activity	-0.09	-0.14/-0.04	-0.02/-0.03	-0.06/0.06
Family consistency in beliefs about screen media	-0.06	-0.02/0.11	**-0.21/-0.22**	0.10/-0.03

^1^correlations appearing in bold indicate significant associations (p<0.05)

Fewer significant associations were observed between Control of Screen Media scales and children’s outdoor playtime and screen time. Having stricter limits and supervision of screen media was negatively associated with screen use (r = -0.18 to -0.50), which could indicate that limits are somewhat effective, more so for TV than video game play. Again, these relationships were generally consistent with what was hypothesized. Parental perceptions of their screen media influence was also negatively associated with children’s TV time (r = -0.32 to -0.35), weekend video game time (r = -0.26), and child BMI percentile (r = -0.14). In contrast, monitoring use and using video games as a bribe was positively associated with video game use (r = 0.26 to 0.28).

Associations between Explicit Modeling scales and children’s outdoor playtime and screen time generally suggested that when parents model behaviors more frequently they are reflected in their children’s behaviors, which was also consistent with hypotheses. Co-participation in physical activity, encouragement for outside play, and facilitation of sports and lessons were all positively associated with children’s outdoor playtime (r = 0.17 to 0.33). Encouragement for outside play was also negatively associated with child BMI percentile (r = -0.25). Meanwhile, co-viewing TV and co-use of video games and computer were positively associated with children’s TV viewing and video game use, respectively (r = 0.33 to 0.44). Co-use of video games and computer was also positively associated with child BMI percentile (r = 0.15).

For Implicit Modeling, parent’s values associated with screen related behaviors showed stronger relationships with children’s screen time compared to physical activity value and children’s outdoor playtime as hypothesized. The value of TV for parent, value of child screen media, and entertainment and education benefits of child screen media were all positively associated with children’s TV and/or video game time (r = 0.18 to 0.39), while only health benefits of physical activity was positively associated with outdoor play time (r = 0.23 to 0.27). Value of parent physical activity and value of child physical activity were negatively associated with child BMI (r = -0.20 for both).

Perceived Facilitators and Barriers scales showed some significant associations with children’s TV viewing. Child preference for inactivity and lack of self-efficacy to limit screen media were positively associated children’s TV viewing (r = 0.19 to 0.37). Enforcement of screen media rules by other adults and family consistency in beliefs about screen media were negatively associated with children’s TV viewing (r = -0.21 to -0.38). Results also showed that lack of support for physical activity from other adults, lack of self-efficacy to limit screen media, and permissiveness for screen media by other adults were positively associated with child BMI percentile (r = 0.14 to 0.17). Relationships were generally in the hypothesized direction.

### Integrated conceptual model

**Fig 1:**
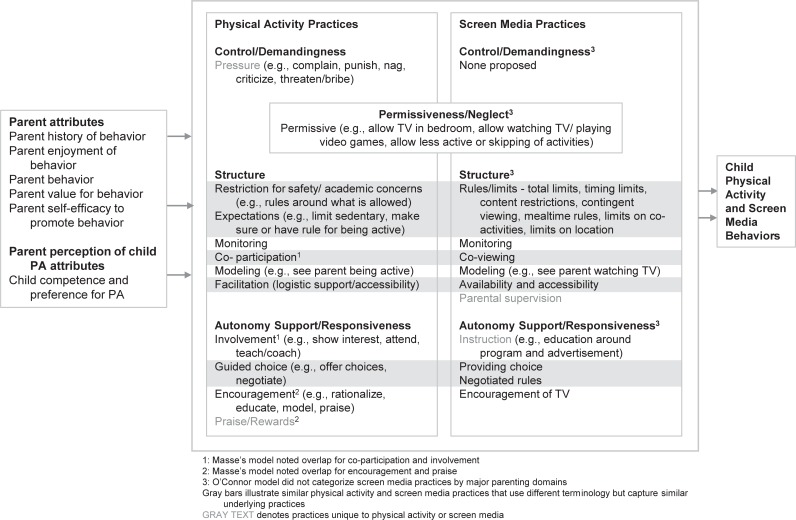
Conceptual model of parent physical activity and screen media practices and beliefs.

An integrated conceptual model of parent physical activity and screen media practices was developed to guide naming of these HomeSTEAD scales and to understand how well final scales capture relevant constructs. Conceptual models by Davison et al. [29] and O’Connor et al. [38] represent some of the first efforts to unite the field around common terminology and definitions. Davison’s conceptual model focuses on physical activity parenting, while O’Connor’s model focuses on screen media. More recently, Masse et al. have published a physical activity conceptual model derived from content mapping of experts’ sorting of parenting practices [37]. Fig 1 illustrates our effort to integrate these conceptual models, apply consistent terminology, and illustrate overlapping versus unique constructs between physical activity and screen media practices and beliefs. Table 4 demonstrates the alignment between this integrated conceptual model and the scales measured in HomeSTEAD. A comparison between the integrated conceptual model and HomeSTEAD’s final scales demonstrates the usefulness of the HomeSTEAD instrument. It captures six physical activity practices (and seven beliefs) aligning with six of the 11 constructs in the model as well as nine screen media practices (and 10 beliefs) aligning with seven of the 11 constructs in the model. (Note: Permissiveness is counted as a screen media practice even through it spans across both physical activity and screen media practices in the integrated conceptual model).

**Table 4:** Conceptual model components compared to constructs captured by HomeSTEAD.

Integrated Conceptual Model Constructs	HomeSTEAD Physical Activity Scales	HomeSTEAD Screen Media Scales
***Parent Attributes***		
Parent history of behavior	*not captured*	*not captured*
Parent enjoyment of behavior	*captured as part of “Parent value for behavior”*	*not captured*
Parent behavior	*not captured*	*not captured*
Parent value for behavior	• Value of parent physical activity • Value of child sports • Value of child physical activity • Health benefits of child physical activity	• Value of TV for parent • Value of child screen media • Entertainment and education benefits of child screen media
Parent self-efficacy to promote behavior	• Perceived influence on physical activity • Lack of support for physical activity from adults • Weather-related barriers to physical activity	• Perceived influence on screen media use • Lack of self-efficacy for limiting screen media • Permissiveness for TV viewing by other adults • Permissiveness for screen media by other adults • Enforcement of screen media rules by other adults • Family consistency in beliefs about screen media
***Parent Perception of Child Physical Activity Attributes***		
Child competence and preference for physical activity	*captured as part of “Child preference for inactivity”*	• Child preference for inactivity
***Control/Demandingness***		
Pressure^PA^ (e.g., pressure, complain, punish, nag, criticize, threaten/ bribe)	• Use of physical activity as a bribe	• Monitoring and use of TV as a threat or bribe • Monitoring and use of video games as a threat or bribe • Use of computers as a threat or bribe
***Permissiveness/Neglect/Indulgence***		
Permissive	• Context driven permissiveness for screen media *also captured as part of “Limits on and supervision of screen media” and in HomeSTEAD’s physical activity and media inventory (not analyzed here)*	
***Structure***		
Restriction for safety or academic concerns^PA^ (e.g., rules around what is allowed)	• Weather-related restriction of outdoor play • Restriction of active play indoors	*not applicable*
Expectations^PA^ (e.g., limit sedentary, make sure or have rule for being active)	*not captured*	*not applicable*
Rules/limits^SM^ (e.g., total limits, content restrictions, contingent viewing, mealtime rules, limits on co-activities, limit on locations)	*not applicable*	• Limits on and supervision of screen media
Monitoring	*not captured*	*captured as part of “Monitoring and use of TV as threat or bribe” and “Monitoring and use of video games as threat or bribe”*
Co-participation^PA^	• Co-participation in physical activity	*not applicable*
Co-viewing^SM^	*not applicable*	• Co-viewing TV • Co-use of video games and computer
Modeling (e.g., child sees parent physical activity or screen media use)	*not captured*	*not captured*
Facilitation^PA^ (e.g., logistic support/ accessibility)	• Facilitation of sports and lessons	*not applicable*
Availability and accessibility^SM^	*not applicable*	*captured as part of HomeSTEAD’s physical activity and media inventory (not analyzed here)*
Parental supervision^SM^	*not applicable*	*captured as part of “Limits on and supervision of screen media”*
***Autonomy Support/Responsiveness***		
Involvement^PA^	*overlaps with “Co-participation in physical activity”*	*not applicable*
Instruction^SM^ (e.g., education)	*not applicable*	*not captured*
Guided choice^PA^ (e.g., offer choices, negotiate)	*not captured*	*not applicable*
Providing choice^SM^	*not applicable*	*not captured*
Negotiated rules^SM^	*not applicable*	• Negotiation of screen media rules
Encouragement (e.g., rationalize, educate, model, praise)	• Encouragement for outside play	• Education and encouragement to reduce screen media
Praises/rewards^PA^	*not captured*	*not applicable*

^PA^ superscripts indicate constructs relevant to only physical activity

^SM^ superscripts indicate constructs relevant to only screen media.

## Discussion

HomeSTEAD’s Family Physical Activity and Screen Media Practices and Beliefs survey was able to capture 32 unique scales that reflect parent practices and beliefs around children’s physical activity and screen media. The survey included seven physical activity and ten screen media beliefs as well as six physical activity and nine screen media practices. Final scales demonstrated good internal consistency, test-retest reliability, and construct validity. The median Cronbach’s alpha was 0.81 (IQR = 0.74–0.85), the median ICC was 0.70 (IQR = 0.66–0.78), and correlations between scale score and children’s behaviors were generally in the expected direction (and stronger for related scales and behaviors, e.g., physical activity practices and child outside play time, screen media practices and child TV time). This final instrument responds well to an identified need for comprehensive measures [30, 31] as it may represent the most comprehensive assessment of parents’ practices and beliefs around physical activity and screen media use. Further, scale reduction allows these constructs to be assessed with efficiency using 114 items. Such a survey could be completed in approximately 15–20 minutes by most individuals. Hence, HomeSTEAD should help to advance the measurement of these constructs and aid in the understanding of how parent beliefs influence parent practices and ultimately child behaviors, thus contributing to the development of interventions to improve physical activity and reduce sedentary time.

There is a variety of existing measures available to assess parents’ beliefs about physical activity and screen media and the strategies they use to encourage those behaviors in their children. According to a 2013 review of measures [30], one of the earliest and most commonly used instruments is the Parental Support for Physical Activity Survey [48]. Using terminology from the integrated conceptual model, the five items in this survey reflect early attempts to assess co-participation, facilitation, education, involvement, and encouragement around child physical activity. These constructs continue to be important, and measures developed since have refined construct assessment. For example, the Activity Support Survey [49] provided two multi-item scales that refined the assessment of co-participation and facilitation.

There have been a handful of more recently developed measures that have begun to assess a greater variety of constructs. One such measure is the Parental Support and Control for Physical Activity Survey [50], a precursor to HomeSTEAD developed by the same research team. This survey includes six scales assessing “controlling” practices, such as restriction/rules and limits and threats and bribes for both physical activity and screen media, as well as eight scales assessing “supportive” practices such as co-participation, modeling, facilitation, and encouragement for physical activity and screen media. Another recent advancement is the Preschooler Physical Activity Parenting Practices Survey [51]. This survey captures three scales assessing “encouraging” practices and four scales capturing “discouraging” practices. Individual items capture pressure, permissiveness, restriction, co-participation, modeling, facilitation, education, and encouragement; however, constructs are not always captured in discrete scales. For example, the 15-item scale labeled as “parent engagement and structure” appears to merge several constructs such as co-participation, modeling, facilitation, education, and encouragement. These examples of existing measures call attention to the difficulty of defining and measuring discrete parent practice constructs.

A comparison between the conceptual model’s physical activity practices and HomeSTEAD measured constructs demonstrates that HomeSTEAD captures six of the 11 identified constructs. Some gaps remain due to the failure for items to come together as an independent factor in the EFA models. For example, the original survey included several modeling-related items (e.g., How often does your child see you (parent) doing (or going to do) something that is physically active?) While items grouped together in the EFA, they failed to load significantly.

Among the constructs captured by HomeSTEAD scales, alignment between constructs and scales was not always one-to-one. For example, the construct restriction aligned with two scales from HomeSTEAD, specifically weather-related restriction of outdoor play and restriction of active play indoors. We also observed multiple constructs merging into a single scale. Specifically, co-participation in physical activity included items that captured constructs of co-participation as well as involvement. Researchers may need to accept that, while conceptually distinct, constructs can be highly related and very likely influenced by the same actions, evaluations, and underlying belief structures of the parents. The inter-relationship between concepts may not allow for distinct constructs to be operationalized with a simple questionnaire. Having distinct constructs may also not be necessary for behavioral development, intervention implementation, or behavioral change as these parent practices are not used in isolation.

A comparison between the conceptual model’s screen media practices and HomeSTEAD’s measured constructs demonstrates that HomeSTEAD captures nine screen media practices which align with seven of the 11 constructs identified in the conceptual model. Some HomeSTEAD scales represent an expansion of the conceptual model–adding new constructs not previously captured. However, other scales suggest simplification of the conceptual model and consolidation of similar constructs. One example of model expansion is the identification of several screen media practices that reflect parents use of control/demandingness, specifically their use of TV, video games, and computer as a threat or bribe. Another example of model expansion is the identification of capture context driven permissiveness (e.g., situations in which parents allow unsupervised screen media use so that they can accomplish other goals). A similar idea of context driven practices has been identified in the feeding practices literature (i.e., context driven provision of snacks) [52]. In contrast, HomeSTEAD scales suggested possible consolidation of the numerous constructs related to rules and limits on children’s screen media. O’Connor et al. identified eight constructs that reflected rule or limits placed on screen media, which have been measured largely by individual items (not scales) [31]. While HomeSTEAD included several of these individual items, they emerged from the EFA as a single factor. Overall, HomeSTEAD captured most constructs from the conceptual model, especially when other sections of HomeSTEAD are also considered. For example, some constructs are captured in HomeSTEAD’s physical activity and media inventory (e.g., constructs of availability and accessibility of screen media) and its family feeding practices survey (e.g., mealtime rules about TV) [34, 53].

Permissiveness remains a construct that would benefit from additional examination. While Masse et al. included permissiveness in their conceptual model of physical activity practices, it was operationalized as not allowing TV in the child’s bedroom, allowing the child to watch TV or play video games whenever he/she wants, and allowing the child to be less active or skip activity [37]. Hence, the integrated conceptual model presented here identifies this construct as spanning both physical activity and screen media practices. Even though HomeSTEAD as a whole included several items similar to those proposed by Masse et al., they did not converge into a permissiveness scale in part because items were located in other sections of HomeSTEAD (e.g., location of TVs being in the physical activity and screen media inventory) [34] or because they were subsumed within other scales (e.g., part of limits on and supervision of screen media).

HomeSTEAD applied rigorous development methods to create a comprehensive measure of physical activity and screen media practices, but the process was not without some limitations. At time HomeSTEAD was originally developed, there were no existing conceptual models of physical activity and screen time practices. Such models would have informed item identification. In absence of such models, item development did use, as recommended [39], a multi-phase process that included integrating existing literature, soliciting expert advice, and using cognitive interviews to ensure item clarity.

During the EFA, the sample size also presented a limitation. Recommended practice is to have sample size seven times the number of items, which for this survey would have required a sample of 1,680 families. It was not feasible, given the funding of the study, to have that large a sample. Instead, items had to be sorted prior to analyses. However, two approaches to sorting were examined based on the best available knowledge at the time. Furthermore, to ensure that HomeSTEAD’s scales remain relevant in light of these advancements, an integrated conceptual model was developed and used to identify and name the scales that emerged from HomeSTEAD. Future research is needed in a larger sample to confirm these findings.

Applicability of the screen media use constructs in the current screen media environment is an additional limitation. At the time of questionnaire development and data collection, TV, computers and video games were the most prominent media devices, but as technology had advanced, the types of media devices that children have changed. It will be important for future studies to adapt and asses the constructs in HomeSTEAD as they relate to children’s use of additional devices (e.g., tables and smartphones).

Another limitation is the reliance on self-report to assess parents’ practices. Unlike other components of HomeSTEAD, it was not possible to accurately assess parent practices during home visits as verification of the self-report. Hence, it was not possible to assess criterion validity. However, the three administrations of the survey allowed a thorough examination of reliability. Results showed that a single administration is adequate; however, two administrations may improve estimates of typical practices.

The sample of parents used to collect HomeSTEAD data may limit its generalizability. Most parents were female/mothers; hence, future research is needed to better understand father’s practices. The sample was also predominantly white, higher income (≥$50,000), and well-educated (college degree or higher). To explore the potential impact, ANOVA (GLM) models were used to compare differences in construct score across these demographic variables, with results showing frequent significant differences between parents based on race and income. Results of these analyses are provided in S2 Table. It was beyond the scope of the current study to assess cross-cultural validity, but these preliminary analyses emphasize the importance of exploring such issues in future research.

A final limitation to note is that reducing scales did result in some cross-loadings and some decreases in factor loadings (i.e., five items spread across three scales had loadings drop below 0.40). Analyses used both internal and external criteria to inform item reduction so that the most relevant items were retained. However, when these instances occurred, items were retained in their original scales as they were deemed important in preserving the construct being measured.

## Conclusions

HomeSTEAD’s Family Physical Activity and Screen Media Practices and Beliefs survey is an important advance for measurement and will aid future research and identification of parenting practices that foster healthy physical activity habits in children. Far too many children have largely sedentary lifestyles, and they need parental support and encouragement to increase their participation in physical activity and reduce the time they spend with screen media. Each of the 32 scales measured in this survey likely play a role in these behaviors. Future research, using tools like HomeSTEAD, are needed to better understand the patterns of parent practices and how those ultimately influence children’s physical activity habits.

## Supporting information

S1 File De-identified dataset. (ZIP)

S1 Table Original, unreduced scales and factor loadings. (DOCX)

S2 Table Correlation matrix with final (reduced) scales. (DOCX)
