# Supplementary material for: HomeSTEAD’s physical activity and screen media practices and beliefs survey: Instrument development and integrated conceptual model
Source: PLoS One. 2019 Dec 31;14(12):e0226984. doi: 10.1371/journal.pone.0226984 (PMC6938346; doi:10.1371/journal.pone.0226984)
Supplement: S1 File — (ZIP) [file pone.0226984.s001.zip › HomeSTEAD data/Homestead PA Social Scale_Simple TableV3.docx]

| **FACTOR** | **ITEMS (reduced)** | **PROPOSED FACTOR NAME** | **ITEMS** | **# ITEMS** |
| --- | --- | --- | --- | --- |
| **Control - Physical Activity (16 items)** | | | | |
| ConPAF1 | F1F(R) F1D(R) F1A(R) | Weather-related restriction of outdoor play | **F1F** – How often do you allow your child to play outside if it is wet?  **F1D** – How often do you allow your child to play outside if it is raining?  **F1A** – How often do you allow your child to play outside on cold days? | 3 |
| ConPAF4 | F3B F3G F3E F3C | Restriction of active play indoors | **F3B** – When are these activities allowed when you child is playing inside? Running around  **F3G** – When are these activities allowed when you child is playing inside? Piling up pillows & jumping on them  **F3E** – When are these activities allowed when you child is playing inside? Jumping from a height  **F3C** – When are these activities allowed when you child is playing inside? Chasing | 4 |
| ConPAF2 | F1B F1G F1J F1E | Use of physical activity as a bribe | **F1B** – How often do you take outside time away from your child for bad behavior?  **F1G** – How often do you use sports or physical activities to control your child’s behavior?  **F1J** – How often do you use physical activity as a punishment for bad behavior?  **F1E** – How often do you use sports or physical activities to get your child to do something? | 4 |
| ConPAF3 | B1A B6B F2D(R) | Perceived influence on physical activity | **B1A** – I have influence over how much physical activity my child gets.  **B6B** – I have influence over how much my child play outside.  **F2D** – I have a little control over how much physical activity my child gets. | 3 |
| **Control – Screen Media (26 items)** | | | | |
| CONsedF2 | TVLMWE6(R) TVLMWK6(R) G6A CMPLMWK8(R) CMPLMWE8(R) I7C(R) VGLMWE6(R) VGLMWK6(R) H6A | Limits on and supervision of screen media | **TVLMWK6** – Do you limit the amount of time your child watches TV or videos during the week? If yes, how much?  **TVLMWE6** – Do you limit the amount of time your child watches TV or videos during the weekend? If yes, how much?  **G6A** – How often is your child supervised when watching TV?  **CMPLMWK8** – Do you limit the amount of time your child uses the computer during the week? If yes, how much?  **CMPLMWE8** – Do you limit the amount of time your child uses the computer during the weekend? If yes, how much?  **I7C** – My child is allowed to turn on the computer without permission.  **VGLMWK6** – Do you limit the amount of time your child plays video games during the week? If yes, how much?  **VGLMWE6** – Do you limit the amount of time your child plays video games during the weekend? If yes, how much?  **H6A** – How often is your child supervised when playing video games? | 9 |
| CONsedF3 | G6I G6G G9B G8E | Monitoring and use of TV as threat or bribe | **G6I** – How often do you use TV time to get your child to do something?  **G6G** – How often do you take away TV, video, or movie time as a punishment for bad behavior?  **G9B** – If I do not regulate or guide my child’s TV watching, then s/he would watch too much.  **G8E** – If I did not monitor my child’s TV viewing, s/he would watch too much TV. | 4 |
| CONsedF4 | H8D H9C H6H H6I | Monitoring and use of VG as threat or bribe | **H8D** – If I do not regulate or guide my child’s video game play s/he would play too much.  **H9C** – If I did not monitor my child’s video game play s/he would play too much.  **H6H** – How often do you use video game time to get your child to do something?  **H6I** – How often do you use video games to control your child’s behavior? | 4 |
| CONsedF5 | I6F I6E H6F | Use of computer as threat or bribe | **I6F** – How often do you use computer time to get your child to do something?  **I6E** – How often do you take away computer time as a punishment for bad behavior?  **H6F** – How often do you offer video game time (handheld or console) as a reward for good behavior? | 3 |
| CONsedF6 | J1CCAT2 J1ACAT2 J1BCAT2 | Negotiation of screen media rules | **J1CCAT2** – Who is responsible for enforcing rules related to TV viewing video game playing or computer use?  **J1ACAT2** – Who is responsible for setting or making rules related to TV viewing video game playing or computer use?  **J1BCAT2** – Who is responsible for deciding when your child can watch TV, play video games, or use the computer? | 3 |
| CONsedF1 | B6F B6C B1B | Perceived influence on screen media use | **B6F** – I have influence over how much my child watches television, plays video games, and uses the computer.  **B6C** – I have influence over how much my child play video games.  **B1B** – I have influence over how much television my child watches. | 3 |
| **Explicit Modeling and Instrumental Support (24 items)** | | | | |
| ExModelF2 | C1D C1C D2D | Co-participation in Physical Activity | **C1D** – How often does your family play outdoors as a form of family recreation?  **C1C** – How often do your family use sport/physical activity as a form of family recreation?  **D2D** – How often do you or another adult in the household start a physically active game with your child? | 3 |
| ExModelF6 | D6I D6J D4C | Encouragement for outside play | **D6I** – During a typical week, how often do you or another adult in the household encourage your child to play outside?  **D6J** – During a typical week, how often do you or another adult in the household try to get your child to play outside when the weather is nice?  **D4C** – How often do you or another adult in the household send your child outside to play? | 3 |
| ExModelF7 | D5A D5B zD3 | Facilitation of sports and lessons | **D5A** – How often in the past 7 days did you or another adult in the household watch your child’s sporting events, lessons, or other organized physical activities with them?  **D5B** – How often in the past 7 days did you or another adult in the household take your child to practice, lessons, classes, or other programs that involve moderate or vigorous physical activity?  **D3** – During the past year, has an adult in your household enrolled your child in lessons, classes, or sports involving moderate or vigorous physical activity? | 3 |
| ExModelF1 | D7B D7C D6c | Encouragement and education to reduce screen media | **D7B** – How often do you or another adult in the household discuss with your child how sedentary habits can be unhealthy?  **D7C** – How often do you or another adult in the household discuss with your child how watching too much TV can be unhealthy?  **D6C** – During a typical week, how often do you or another adult in the household say things to encourage your child to spend less time being sedentary? | 3 |
| ExModelF4 | D4A C1B G6C D6E | Co-viewing of TV | **D4A** – How often do you or another adult in the household watch TV with your child?  **C1B** – How often does your family watch TV or movies as a form of family recreation?  **G6C** – How often does your child see you or another adult in the household watching TV/movies?  **D6E** – During a typical week how often do you or another adult in the household ask your child to watch TV with you? | 4 |
| ExModelF3 | D4E C1A D6G | Co-use of video games and computer | **D4E** – How often do you or another adult in the household play video games with your child?  **C1A** – How often does your family play video games as a form of family recreation?  **D6G** – During a typical week, how often do you or another adult in the household use the computer with your child? | 3 |
| ExModelF5 | D2e G8f I8d H7b H7e | Context driven permissiveness for screen media | **D2E** – How often do you or another adult in the household turn on the TV, a video, or movie for your child so you can get things done around the house?  **G8F** – When my child watches TV it helps me get things done around the house.  **I8D** – When my child uses the computer it helps me get things done around the house.  **H7B** – When my child plays video games, it helps me get things done around the house.  **H7E** – When my child is bored it helps to turn on a video game. | 5 |
| **Implicit Modeling (23 items)** | | | | |
| ImModelF4 | A3B A1A A1E A2A(R) | Value of parent Physical Activity | **A3B** – Participating in physical activity is valuable to me.  **A1A** – Participating in regular physical activity is important to me.  **A1E** – I look forward to being physical active.  **A2A** – I do not enjoy being physically active in my free time. | 4 |
| ImModelF7 | E3B E2B | Value of child sports | **E3B** – My child benefits from playing sports.  **E2B** – How important is it that your child participates in organized sports and activities? | 2 |
| ImModelF6 | E5B E3A E2A | Value of child Physical Activity | **E5B** – Children who do regular physical activity are more healthy.  **E3A** – My child benefits from being physically active.  **E2A** – How important is it that your child does physical activities in his/her free time? | 3 |
| ImModelF5 | E5F E5D E5E | Health benefits of child Physical Activity | **E5F** – Children who do regular physical activity are less stressed.  **E5D** – Children who do regular physical activity are less likely to be overweight.  **E5E** – Children who do regular physical activity are happier. | 3 |
| ImModelF1 | A1F A3C G9A | Value of TV for parent | **A1F** – Watching TV is important to me.  **A3C** – Watching TV is valuable to me.  **G9A** – Watching TV is good entertainment for my child. | 3 |
| ImModelF2 | E2D E2C E3E B1D | Value of child screen media | **E2D** – How important is it that your child be able to play video games during their free time?  **E2C** – How important is it that your child be able to watch TV or movies during their free time?  **E3E** – My child benefits from using the internet.  **B1D** – I enjoy playing video games with my child. | 4 |
| ImModelF3 | I8A H8C H9B G7A | Entertainment and education benefits of child screen media | **I8A** – Using the computer is good entertainment for my child.  **H8C** – Playing video games is good entertainment for my child.  **H9B** – Playing video games helps my child learn.  **G7A** – Watching TV helps my child learn. | 4 |
| **Perceived Barriers and Facilitators (24 items)** | | | | |
| CPBF1 | E6I E1(R) E4B(R) | Child preference for inactivity | **E6I** – My child’s PA is limited due to my child’s lack of interest or motivation.  **E1** – What does your child usually do when s/he has a choice about how to spend their free time?  **E4B** – When outside, my child prefers: (scale: light play vs. very active play) | 3 |
| CPBF3 | E6G E6A E6B | Lack of support for Physical Activity from adults | **E6G** – My child’s PA is limited due to my own lack of motivation and interest.  **E6A** – My child’s PA is limited due to lack of adult supervision.  **E6B** – My child’s PA is limited due to other adults in my child’s life. | 3 |
| CPBF2 | I9C G7E H7D H9A G9C | Lack of self-efficacy for limiting screen media | **I9C** – It is hard to limit the amount of time my child spends on the computer.  **G7E** – When I am tired it is hard to get my child to watch less TV.  **H7D** – It is hard to limit the amount of video games my child plays.  **H9A** – My child’s begging or nagging makes it difficult to get him/her to play video games less.  **G9C** – It is hard to limit the amount of TV my child watches. | 5 |
| CPBF6 | G8D G7C | Permissiveness for TV viewing by other adults | **G8D** – Other adults in my child’s life make it difficult to enforce household rules about TV viewing  **G7C** – Other adults in my child’s life make it difficult to get my child to watch less TV | 2 |
| CPBF5 | H7A I8B I7A | Permissiveness for screen media by other adults | **H7A** – Other adults in my child’s life make it difficult to get my child to play video games less.  **I8B** – Other adults in my child’s life make it difficult to enforce household rules about computer use.  **I7A** – Other adults in my child’s life make it difficult to get my child to play on the computer less. | 3 |
| CPBF8 | G9F H9E I9E | Enforcement of screen media rules by other adults | **G9F** – Other adults in my household tightly enforce the household rules related to TV viewing.  **H9E** – Other adults in my household tightly enforce the household rules related to video game play/use.  **I9E** – Other adults in my household tightly enforce the household rules related to computer use. | 3 |
| CPBF4 | E6J E6K | Weather-related barriers to Physical Activity | **E6J** – My child’s PA is limited due to cold weather.  **E6K** – My child’s PA is limited due to hot weather. | 2 |
| CPBF7 | J2C J2B J2A | Family consistency in beliefs around screen media | **J2C** – You or another adult in the household have the same views about computer use.  **J2B** – You or another adult in the household have the same views about video game playing.  **J2A** – You or another adult in the household have the same views about computer use. | 3 |
