# Supplementary material for: HomeSTEAD’s physical activity and screen media practices and beliefs survey: Instrument development and integrated conceptual model
Source: PLoS One. 2019 Dec 31;14(12):e0226984. doi: 10.1371/journal.pone.0226984 (PMC6938346; doi:10.1371/journal.pone.0226984)
Supplement: S1 Table — (DOCX) [file pone.0226984.s002.docx]

**S1 Table. Original, unreduced scales and factor loadings**

| **Scale Name and Items** | **Factor loading** |
| --- | --- |
| **Control of Physical Activity** |  |
| ***Weather-related restriction of outdoor play*** |  |
| How often do you allow your child to play outside if it is wet? (R) | 0.90 |
| How often do you allow your child to play outside if it is raining? (R) | 0.83 |
| How often do you allow your child to play outside on cold days? (R) | 0.78 |
| My child can only play in the yard when I or another adult can be outside within him/her. | 0.60 |
| My child can only play in yard where I can see him/her from inside. | 0.58 |
| How often do you allow your child to play outside on hot days? (R) | 0.54 |
| When outside, my child can get to toys or equipment without the help from an adult. (R) | 0.52 |
| When outside, my child has to ask for a toy or equipment before I will get them out. | 0.44 |
| ***Restriction of active play indoors*** |  |
| When are these activities allowed when your child is playing inside?^2^ |  |
| Running around | 0.87 |
| Chasing | 0.79 |
| Hopping, skipping, or galloping | 0.78 |
| Piling up pillows and jumping on them | 0.62 |
| Jumping from a height | 0.61 |
| Rough housing or wrestling | 0.60 |
| Throwing, kicking, or bouncing a ball | 0.53 |
| Flipping (somersault) or tumbling | 0.52 |
| When inside, my child is allowed to use toys and equipment for active play involving gross motor activities like running, jumping, hopping, or tumbling. (R) | 0.61 |
| When my child is inside the house, his/her play should be calm and quiet. | 0.51 |
| When inside, my child can easily get toys that are used for physically active play (R) | 0.30 |
| ***Use of physical activity as a bribe*** |  |
| How often do you take outside time away from your child for bad behavior? | 0.73 |
| How often do you give your child extra outside time as a reward? | 0.73 |
| How often do you use sports or physical activities to control your child’s behavior? | 0.64 |
| How often do you use sports or physical activities to get your child to do something? | 0.64 |
| I ask my child not to get his/her clothes dirty while s/he is playing outside. | 0.40 |
| How often so you use physical activity as a punishment for bad behavior? | 0.39 |
| How often do you keep track of the physical activity your child is getting each day? | 0.39 |
| ***Perceived influence on physical activity*** |  |
| I am in charge over how much physical activity my child gets. | 0.78 |
| I have influence over how much physical activity my child gets. | 0.70 |
| I have influence over how much my child plays outside. | 0.69 |
| I have little control over how much physical activity my child gets. (R) | 0.48 |
| The amount of physical activity I get influences how much my child gets. | 0.36 |
| **Control of Screen Media** |  |
| ***Limits on and supervision of screen media*** |  |
| Do you limit the amount of time your child watches TV or videos during ***weekend***? If yes, how much? (R) | 0.80 |
| Do you limit the amount of time your child watches TV or videos during ***week***? If yes, how much? (R) | 0.65 |
| My child is allowed to turn on the TV without permission. (R) | 0.76 |
| If my child has finished his/her school/house work I do not limit his/her computer time. (R) | 0.75 |
| If my child has finished his/her school/house work I do not limit how much TV s/he watches. (R) | 0.74 |
| Do you limit the amount of time your child uses the computer during ***weekend***? If yes, how much? (R) | 0.71 |
| Do you limit the amount of time your child uses the computer during ***week***? If yes, how much? (R) | 0.72 |
| Do you place limits on the programs your child is allowed to watch on TV? | 0.65 |
| Do you limit the amount of time your child plays video games during ***weekend***? If yes, how much? (R) | 0.62 |
| Do you limit the amount of time your child plays video games during ***week***? If yes, how much? (R) | 0.51 |
| My child is allowed to turn on the computer without permission. (R) | 0.59 |
| If my child has finished his/her school/house work I do not limit video game play. (R) | 0.59 |
| I tightly enforce the household rules related to TV viewing. | 0.51 |
| My child is allowed to turn on the video game system without permission. (R) | 0.49 |
| How often is your child supervised when using the computer? | 0.49 |
| Do you place limits on the video games your child is allowed to play? | 0.47 |
| How often is your child supervised when playing video games? | 0.45 |
| How often is your child supervised when watching TV? | 0.41 |
| ***Monitoring and use of TV as a threat or bribe*** |  |
| How often do you use TV time to get your child to do something? | 0.68 |
| How often do you use TV to control child’s behavior? | 0.60 |
| How often do you offer TV, video, or movie time as a reward for good behavior? | 0.55 |
| How often do you take away TV, video, or movie time as a punishment for bad behavior? | 0.53 |
| If I do not ***regulate or guide*** my child’s TV watching, s/he would watch too much. | 0.52 |
| If I did not ***monitor*** my child’s TV viewing, s/he would watch too much TV? | 0.52 |
| ***Monitoring and use of video games as a threat or bribe*** |  |
| If I do not ***regulate or guide*** my child’s video game play, s/he would play too much. | 0.87 |
| If I did not ***monitor*** my child’s video game play, s/he would play too much? | 0.84 |
| How often do you use video game time to get your child to do something? | 0.74 |
| How often do you take away video game time as a punishment for bad behavior? | 0.67 |
| How often do you use video games to control your child’s behavior? | 0.64 |
| If I do not ***regulate or guide*** my child’s computer use then s/he would use too much. | 0.45 |
| ***Use of computers as a threat or bribe*** |  |
| How often so you use computer time to get your child to do something? | 0.94 |
| How often do you offer computer time to your child as a reward for good behavior? | 0.88 |
| How often do you take away computer time as a punishment for bad behavior? | 0.81 |
| How often do you use computer time to control your child’s behavior? | 0.72 |
| How often do you offer video game time (handheld or console) as a reward for good behavior? | 0.41 |
| If I do not ***monitor*** my child’s computer use, s/he uses the computer too much. | 0.36 |
| ***Negotiation of screen media rules*** |  |
| Who is responsible for enforcing rules related to TV viewing, video game playing, or computer use? | 0.96 |
| Who is responsible for setting rules related to TV viewing, video game playing, or computer use? | 0.93 |
| Who is responsible for deciding when your child can watch TV, play video games, or use the computer? | 0.92 |
| ***Perceived influence on screen media use*** |  |
| I have influence over how much my child watches TV, plays video games, and uses the computer. | 0.91 |
| I am in charge of how much TV, video game, and computer use my child gets. | 0.78 |
| I have influence over how much my child plays video games. | 0.70 |
| I have influence over how much television my child watches. | 0.62 |
| I have influence over how much my child uses the computer. | 0.57 |
| I have very little control over how much TV, video game, and computer my child uses. (R) | 0.55 |

| **Explicit Modeling** |  |
| --- | --- |
| ***Co-participation in physical activity*** |  |
| How often do you or another adult in the household join your child in physically active play? | 0.84 |
| How often do you or another adult in the household play outside with your child? | 0.77 |
| How often does your family play outdoors as a form of family recreation? | 0.68 |
| During a typical week, how often do you or another adult in the household start a game or activity for your child that involves vigorous physical activity? | 0.68 |
| How often do you or another adult in the household start a physically activity game with your child? | 0.66 |
| How often do you or another adult in the household get out a toy or piece of equipment without being asked that will be used by your child during moderate or vigorous physical activity? | 0.54 |
| How often does your family use sport/physical activity as a form of family recreation? | 0.51 |
| How often do you or another adult in the household send your child outside so that you can get things done around the house? (R) | 0.41 |
| How often do you discuss with your child what s/he does on the computer? | 0.36 |
| ***Encouragement for outside play*** |  |
| During a typical week, how often do you or another adult in the household encourage your child to play outside? | 1.0 |
| During a typical week, how often do you or another adult in the household try to get your child to play outside when the weather is nice? | 0.61 |
| How often do you or another adult in the household send your child outside to play? | 0.49 |
| ***Facilitation of sports and lessons*** |  |
| How often in the past 7 days did you or another adult in the household watch your child’s sporting events, lessons, or other organized physical activities with them? | 0.81 |
| How often in the past 7 days did you or another adult in the household take your child to practice, lessons, classes, or other programs that involved moderate or vigorous physical activity? | 0.78 |
| During the past year, has an adult in your household enrolled your child in lessons, classes, or sports involving moderate or vigorous physical activity? | 0.44 |
| During a typical week, how often do you or another adult in the household watch your child participate in physical activity or sport? | 0.44 |
| ***Encouragement and education to reduce screen media*** |  |
| How often do you or another adult in the household discuss with your child how sedentary habits can be unhealthy? | 0.76 |
| How often do you or another adult in the household discuss with your child how being physically activity is good for their health? | 0.76 |
| During a typical week, how often do you or another adult in the household say things to encourage your child to spend less time being sedentary? | 0.75 |
| During a typical week, how often do you or another adult in the household say things to encourage your child to do physical activities? | 0.71 |
| How often do you or another adult in the household discuss with your child how watching too much TV can be unhealthy? | 0.70 |
| How often do you or another adult in the household discuss with your child how being physically active builds strong muscles? | 0.69 |
| During a typical week, how often do you or another adult in the household encourage your child to be physically active? | 0.66 |
| During a typical week, how often do you or another adult in the household tell your child that playing outside is good for their health? | 0.64 |
| How often do you or another adult in the household praise your child for being physically active? | 0.57 |
| How often do you or another adult in the household praise your child for participating in sports? | 0.49 |
| How often do you or another adult in the household say things to encourage your child to spend less time watching TV or movies? | 0.46 |
| ***Co-viewing TV*** |  |
| During a typical week, how many days per week do you or another adult in your household watch TV, videos, or DVDSs together with your child? On these days, about how many minutes per day are watched with you or another adult? | 0.75 |
| How often do you or another adult in the household watch TV with your child? | 0.71 |
| How often does your family watch TV or movies as a form of family recreation? | 0.63 |
| How often does your child see you or another adult in the household watching TV/movies? | 0.47 |
| During a typical week, how often do you or another adult in the household ask your child to watch TV with you? | 0.44 |
| How often do you discuss with your child what s/he sees or does on the video game s/he plays? (R) | 0.35 |
| ***Co-use of video games and computer*** |  |
| How often do you or another adult in your household play video games with your child? | 0.82 |
| How often does your family play video games as a form of family recreation? | 0.74 |
| During a typical week, how many days per week do you or another adult in your household play video games together with your child? On these days, about how many minutes per day are played with you or another adult? | 0.74 |
| During a typical week, how often do you or another adult in your household use the computer with your child? | 0.47 |
| How often does child see you or another adult in your household play video games? | 0.45 |
| During a typical week, how many days per week do you or another adult in your household use the computer with your child? On these days, about how many minutes per day were they on the computer with you or another adult? | 0.36 |
| ***Context driven permissiveness for screen media*** |  |
| How often do you or another adult in the household turn on the TV, video, or a movie for your child so you can get things done around the house? | 0.77 |
| When my child watches TV, it helps me get things done around the house. | 0.74 |
| When my child uses the computer, it helps me get things done around the house. | 0.68 |
| When my child plays video games, it helps me get things done around the house. | 0.59 |
| When my child is bored, it helps to turn on a video game. | 0.52 |
| When my child is bored it helps to turn on the computer. | 0.47 |
| How often do you or another adult in the household turn on the TV or a movie so you can sleep a little longer or get some rest? | 0.34 |
| **Implicit Modeling** |  |
| ***Value of parent physical activity*** |  |
| Participating in physical activity is valuable to me. | 0.90 |
| Participating in physical activity is important to me. | 0.90 |
| I enjoy being physically active in my free time. | 0.87 |
| Participating in regular physical activity is important to me. | 0.81 |
| I find participating in physical activity valuable. | 0.79 |
| I enjoy doing vigorous physical activity. | 0.74 |
| I look forward to being physically active. | 0.74 |
| I do not enjoy being physically active in my free time. (R) | 0.71 |
| I enjoy doing vigorous physical activity with my child. | 0.38 |
| I enjoy being physically active with my child. | 0.38 |
| ***Value of child sports*** |  |
| My child benefits from playing sports. | 0.91 |
| How important is it that your child participates in organized sports and activities? | 0.59 |
| ***Value of child physical activity*** |  |
| Children who do regular physical activity are more healthy. | 0.77 |
| My child benefits from being physically active. | 0.74 |
| How important is it that your child does physical activities in his/her free time? | 0.43 |
| My child benefits from playing outside. | 0.37 |
| ***Health benefits of child physical activity*** |  |
| Children who do regular physical activity are less stressed. | 0.89 |
| Children who do regular physical activity keep from gaining too much weight. | 0.86 |
| Children who do regular physical activity are less likely to be overweight. | 0.81 |
| Children who do regular physical activity will be healthy adults. | 0.61 |
| Children who do regular physical activity are happier. | 0.59 |
| Children who do regular physical activity have more self-confidence. | 0.53 |
| ***Value of TV for parent*** |  |
| Watching TV is important to me. | 0.91 |
| Watching TV is valuable to me. | 0.83 |
| I enjoy watching TV or movies during my free time. | 0.57 |
| Watching TV is good entertainment for my child. | 0.52 |
| I enjoy playing video games during my free time. | 0.39 |
| Watching TV takes time away from more important things. (R) | 0.34 |
| ***Value of child screen media*** |  |
| How important is it that your child be able to play video games during their free time? | 0.72 |
| How important is it that your child be able to watch TV or movies during their free time? | 0.67 |
| My child benefits from playing video games. | 0.65 |
| I enjoy playing video games with my child. | 0.53 |
| I enjoy using the computer with my child. | 0.49 |
| My child benefits from watching TV or videos. | 0.49 |
| My child benefits from using the internet. | 0.47 |
| I enjoy watching TV or movies with my child. | 0.47 |
| ***Entertainment and education benefits of child screen media*** |  |
| Using the computer is good entertainment for my child. | 0.80 |
| Playing video games is good entertainment for my child. | 0.75 |
| Using the computer helps my child learn. | 0.71 |
| Playing video games helps my child learn. | 0.65 |
| Watching TV helps my child learn. | 0.36 |
| **Perceived Barriers and Facilitators** |  |
| ***Child preference for inactivity*** |  |
| When outside, my child prefers… (light play ↔ very active play) (R) | 0.60 |
| My child’s physical activity is limited due to my busy schedule. | 0.52 |
| I think it is difficult to encourage my child to go outside and play. | 0.52 |
| My child’s physical activity is limited due to my child’s lack of interest or motivation. | 0.51 |
| What does your child usually do when s/he has a choice about how to spend his/her free time? (R) | 0.51 |
| ***Lack of support for physical activity from adults*** |  |
| My child’s physical activity is limited due to lack of adult supervision. | 0.65 |
| My child’s physical activity is limited due to safety of my neighborhood. | 0.61 |
| My child’s physical activity is limited due to the size of my yard. | 0.60 |
| My child’s physical activity is limited due to lack of support from family, spouse, or friends. | 0.53 |
| My child’s physical activity is limited due to my own lack of motivation and interest. | 0.47 |
| My child’s physical activity is limited due to fees for clubs, activities, or facilities being too high. | 0.47 |
| My child’s physical activity is limited due to other adults in my child’s life. | 0.44 |
| ***Lack of self-efficacy for limiting screen media*** |  |
| It is hard to limit the amount of time my child spends on the computer. | 0.88 |
| When I am tired it is hard to get my child to watch less TV. | 0.81 |
| When I am tired it is hard to get my child to play video games less. | 0.78 |
| My child’s begging or nagging makes it difficult to get him/her to watch less TV. | 0.75 |
| My child’s begging or nagging makes it difficult to get him/her to play on the computer less. | 0.74 |
| It is hard to limit the amount of video games my child plays. | 0.74 |
| My child’s begging or nagging makes it difficult to get him/her to play video games less. | 0.71 |
| It is hard to limit the amount of TV my child watches. | 0.71 |
| When I am tied it is hard to get my child to play on the computer less. | 0.63 |
| Other adults in my child’s life make it difficult to enforce household rules about video game play. | 0.52 |
| When inside, my child prefers… (light play ↔ very active play) | 0.38 |
| ***Permissiveness for TV viewing by other adults*** |  |
| Other adults in my child’s life make it difficult to enforce household rules about TV viewing. | 0.69 |
| Other adults in my child’s life make it difficult to get my child to watch less TV. | 0.68 |
| ***Permissiveness for screen media by other adults*** |  |
| Other adults in my child’s life make it difficult to enforce household rules about computer use. | 0.89 |
| Other adults in my child’s life make it difficult to get my child to play on the computer less. | 0.85 |
| Other adults in my child’s life make it difficult to get my child to play video games less. | 0.44 |
| ***Enforcement of screen media rules by other adults*** |  |
| Other adults in my household tightly enforce the household rules related to video game play/use. | 0.70 |
| Other adults in my household tightly enforce the household rules related to computer use. | 0.62 |
| Other adults in my household tightly enforce the household rules related to TV viewing. | 0.50 |
| ***Weather-related barriers to physical activity*** |  |
| My child’s physical activity is limited due to cold weather. | 0.94 |
| My child’s physical activity is limited due to hot weather. | 0.71 |
| ***Family consistency in beliefs about screen media*** |  |
| You or another adult in the household have the same views about computer use. | 0.97 |
| You or another adult in the household have the same views about video game playing. | 0.81 |
| You or another adult in the household have the same views about how much TV child should watch | 0.79 |
