# Supplementary material for: HomeSTEAD’s physical activity and screen media practices and beliefs survey: Instrument development and integrated conceptual model
Source: PLoS One. 2019 Dec 31;14(12):e0226984. doi: 10.1371/journal.pone.0226984 (PMC6938346; doi:10.1371/journal.pone.0226984)
Supplement: S2 Table — (DOCX) [file pone.0226984.s003.docx]

**S2 Table. Correlation matrix with final (reduced) scales.**

|  |  | **1** | **2** | **3** | **4** | **5** | **6** | **7** | **8** | **9** | **10** | **11** | **12** | **13** | **14** | **15** | **16** |
| --- | --- | --- | --- | --- | --- | --- | --- | --- | --- | --- | --- | --- | --- | --- | --- | --- | --- |
| 1 | Weather-related restriction of outdoor play | 1 |  |  |  |  |  |  |  |  |  |  |  |  |  |  |  |
| 2 | Restriction of active play indoors | **0.22** | 1 |  |  |  |  |  |  |  |  |  |  |  |  |  |  |
| 3 | Use of physical activity as a bribe | 0.14 | **0.21** | 1 |  |  |  |  |  |  |  |  |  |  |  |  |  |
| 4 | Perceived influence on physical activity | -0.09 | -0.04 | -0.09 | 1 |  |  |  |  |  |  |  |  |  |  |  |  |
| 5 | Limits on and supervision of screen media | -0.04 | 0.12 | 0.08 | **-0.2** | 1 |  |  |  |  |  |  |  |  |  |  |  |
| 6 | Monitoring and use of TV as a threat or bribe | 0.03 | 0.05 | **0.38** | 0.13 | -0.07 | 1 |  |  |  |  |  |  |  |  |  |  |
| 7 | Monitoring and use of video games as a threat or bribe | -0.09 | 0.09 | **0.36** | 0.05 | -0.09 | **0.59** | 1 |  |  |  |  |  |  |  |  |  |
| 8 | Use of computer as threat or bribe | **0.18** | **0.22** | **0.53** | -0.1 | 0.02 | **0.43** | **0.54** | 1 |  |  |  |  |  |  |  |  |
| 9 | Negotiation of screen media rules | 0.07 | 0.11 | **0.19** | 0.03 | 0.03 | 0 | -0.04 | 0.01 | 1 |  |  |  |  |  |  |  |
| 10 | Perceived influence on screen media use | -0.07 | -0.09 | -0.09 | **0.57** | **-0.43** | **0.19** | 0.09 | -0.02 | 0.03 | 1 |  |  |  |  |  |  |
| 11 | Co-participation in physical activity | **-0.16** | **-0.15** | 0.08 | 0.15 | **-0.28** | -0.01 | 0.08 | -0.02 | 0.1 | **0.18** | 1 |  |  |  |  |  |
| 12 | Encouragement for outside play | **-0.37** | **-0.17** | 0.04 | **0.3** | -0.13 | **0.2** | **0.26** | -0.09 | -0.02 | **0.21** | **0.33** | 1 |  |  |  |  |
| 13 | Facilitation of sports and lessons | -0.1 | -0.08 | 0.01 | 0.02 | -0.07 | 0 | 0.07 | -0.02 | -0.08 | 0.04 | **0.28** | 0.1 | 1 |  |  |  |
| 14 | Encouragement fand education to reduce screen media | -0.06 | -0.02 | 0.11 | 0.15 | -0.06 | **0.32** | **0.39** | 0.13 | 0.04 | 0.12 | **0.2** | **0.4** | **0.24** | 1 |  |  |
| 15 | Co-viewing of TV | **0.2** | **0.23** | **0.27** | -0.09 | **0.45** | 0.13 | -0.09 | **0.16** | -0.01 | **-0.2** | 0.02 | -0.08 | 0.06 | **0.17** | 1 |  |
| 16 | Co-use of video games and computer | 0.1 | 0.07 | **0.26** | -0.09 | **0.3** | 0.04 | **0.17** | 0.12 | **0.15** | -0.11 | **0.19** | -0.05 | 0.14 | **0.17** | **0.42** | 1 |
| 17 | Context driven permissiveness for screen media | 0.03 | -0.01 | 0.09 | -0.1 | 0.02 | **0.31** | **0.18** | **0.29** | **-0.17** | **-0.21** | 0.06 | 0.13 | -0.03 | 0.05 | 0.06 | -0.04 |
| 18 | Value of parnet physical activity | **-0.21** | -0.14 | -0.09 | 0.12 | 0.05 | 0.03 | 0.06 | **-0.2** | 0.08 | 0.07 | **0.29** | **0.2** | **0.24** | **0.24** | -0.06 | 0.01 |
| 19 | Value of child sports | 0.09 | 0 | **0.16** | 0.12 | 0.1 | **0.17** | -0.01 | 0.09 | 0.04 | 0.06 | 0.08 | 0.06 | **0.4** | **0.21** | **0.27** | 0.1 |
| 20 | Value of child physical activity | **-0.23** | **-0.18** | -0.07 | **0.48** | -0.14 | **0.18** | 0.11 | -0.09 | 0 | **0.36** | **0.26** | **0.41** | **0.16** | **0.21** | -0.13 | -0.13 |
| 21 | Health benefits of child physical activity | **-0.19** | **-0.17** | -0.01 | **0.33** | -0.12 | 0.13 | 0.09 | -0.03 | 0 | **0.27** | **0.18** | **0.31** | 0.12 | **0.24** | -0.05 | -0.1 |
| 22 | Value of TV for parent | 0.08 | 0.12 | 0.07 | **-0.17** | **0.3** | 0.11 | **-0.16** | 0.03 | -0.15 | **-0.3** | -0.09 | -0.09 | 0 | -0.12 | **0.39** | 0.09 |
| 23 | Value of child screen media | 0.15 | **0.2** | **0.21** | **-0.23** | **0.4** | -0.02 | 0 | 0.1 | 0.1 | **-0.32** | 0.07 | -0.06 | **0.16** | 0.11 | **0.48** | **0.55** |
| 24 | Entertainment and education benefits of child screen media | 0.05 | -0.06 | -0.03 | -0.01 | **0.2** | -0.07 | 0 | 0.05 | -0.15 | -0.15 | 0.07 | 0.1 | 0 | -0.08 | 0.12 | 0.12 |
| 25 | Child preference for inactivity | 0 | 0.06 | -0.11 | **-0.34** | 0.07 | 0.07 | -0.03 | 0.03 | -0.14 | -0.08 | -0.12 | **-0.16** | -0.07 | -0.13 | 0.06 | -0.12 |
| 26 | Lack of support for physical activity from adults | 0.13 | 0.07 | 0.07 | **-0.15** | -0.04 | **0.19** | 0 | **0.25** | **-0.19** | -0.12 | -0.13 | -0.13 | **-0.16** | -0.12 | 0.1 | -0.13 |
| 27 | Lack of self-efficacy for limiting screen media | -0.1 | -0.02 | 0.11 | **-0.32** | **0.25** | **0.29** | **0.22** | **0.31** | **-0.3** | **-0.41** | 0 | -0.05 | 0 | 0.03 | **0.15** | 0.06 |
| 28 | Permissiveness for TV viewing by other adults | -0.12 | -0.04 | -0.01 | **-0.2** | 0.08 | **0.19** | 0.03 | 0.12 | **-0.24** | -0.14 | 0.01 | 0.01 | 0.05 | -0.03 | 0.08 | 0.07 |
| 29 | Permissiveness for screen media by other adults | 0 | 0.06 | 0.12 | **-0.25** | 0.05 | **0.19** | **0.21** | **0.25** | **-0.22** | **-0.2** | 0.01 | -0.03 | 0.01 | -0.03 | 0.02 | 0.1 |
| 30 | Enforcement of screen media rules by other adults | 0.01 | -0.13 | 0.01 | **0.29** | **-0.46** | 0.13 | **0.21** | -0.04 | 0.1 | **0.39** | **0.22** | **0.26** | 0.06 | **0.18** | **-0.24** | 0.05 |
| 31 | Weather-related barriers to child physical activity | **0.23** | -0.13 | -0.1 | -0.05 | **-0.18** | 0.03 | -0.01 | -0.02 | -0.09 | 0.03 | 0.15 | 0 | 0.09 | 0.1 | -0.01 | -0.03 |
| 32 | Family consistency in beliefs around screen media | -0.06 | -0.15 | 0.05 | **0.17** | **-0.22** | -0.06 | 0.12 | -0.03 | **0.16** | **0.23** | **0.19** | **0.18** | 0.09 | 0.11 | -0.14 | -0.02 |

Correlations in red are significant (p<0.05)

continued…

|  |  | **17** | **18** | **19** | **20** | **21** | **22** | **23** | **24** | **25** | **26** | **27** | **28** | **29** | **30** | **31** |
| --- | --- | --- | --- | --- | --- | --- | --- | --- | --- | --- | --- | --- | --- | --- | --- | --- |
| 17 | Context driven permissiveness for screen media | 1 |  |  |  |  |  |  |  |  |  |  |  |  |  |  |
| 18 | Value of parnet physical activity | 0.03 | 1 |  |  |  |  |  |  |  |  |  |  |  |  |  |
| 19 | Value of child sports | -0.09 | 0.13 | 1 |  |  |  |  |  |  |  |  |  |  |  |  |
| 20 | Value of child physical activity | 0.03 | **0.37** | **0.33** | 1 |  |  |  |  |  |  |  |  |  |  |  |
| 21 | Health benefits of child physical activity | 0.01 | **0.3** | **0.25** | **0.66** |  |  |  |  |  |  |  |  |  |  |  |
| 22 | Value of TV for parent | **0.33** | 0.02 | 0.11 | **-0.16** | -0.15 | 1 |  |  |  |  |  |  |  |  |  |
| 23 | Value of child screen media | 0.14 | 0 | **0.22** | **-0.2** | **-0.18** | **0.39** | 1 |  |  |  |  |  |  |  |  |
| 24 | Entertainment and education benefits of child screen media | **0.4** | **0.2** | -0.06 | 0.07 | -0.06 | **0.3** | **0.33** | 1 |  |  |  |  |  |  |  |
| 25 | Child preference for inactivity | **0.16** | -0.13 | **-0.24** | **-0.28** | **-0.19** | 0.04 | 0.02 | **0.21** | 1 |  |  |  |  |  |  |
| 26 | Lack of support for physical activity from adults | **0.32** | -0.14 | -0.12 | -0.1 | -0.05 | 0.08 | -0.11 | **0.19** | **0.35** | 1 |  |  |  |  |  |
| 27 | Lack of self-efficacy for limiting screen media | **0.55** | 0.04 | -0.01 | -0.08 | -0.08 | **0.35** | **0.17** | **0.26** | **0.26** | **0.38** | 1 |  |  |  |  |
| 28 | Permissiveness for TV viewing by other adults | **0.36** | 0.02 | 0.07 | -0.02 | -0.02 | **0.2** | 0.06 | **0.16** | **0.18** | **0.45** | **0.53** | 1 |  |  |  |
| 29 | Permissiveness for screen media by other adults | **0.34** | -0.04 | 0 | -0.1 | -0.1 | **0.23** | 0.11 | 0.1 | 0.14 | **0.37** | **0.59** | **0.68** | 1 |  |  |
| 30 | Enforcement of screen media rules by other adults | -0.12 | 0.09 | -0.08 | **0.25** | 0.11 | **-0.32** | **-0.17** | -0.05 | -0.11 | -0.1 | **-0.28** | **-0.23** | **-0.27** | 1 |  |
| 31 | Weather-related barriers to child physical activity | 0.11 | 0.08 | -0.06 | 0.02 | -0.08 | 0.03 | -0.1 | **0.2** | **0.19** | **0.28** | 0.12 | 0.09 | 0.12 | 0.1 | 1 |
| 32 | Family consistency in beliefs around screen media | -0.1 | 0.01 | -0.03 | **0.18** | 0.11 | **-0.23** | -0.09 | 0.05 | -0.08 | **-0.3** | **-0.33** | **-0.47** | **-0.49** | **0.49** | -0.03 |

Correlations in red are significant (p<0.05)

**Supplemental Material: Results from simple ANOVA (GLM) models comparing differences in scale scores across demographic variable. Numbers are resulting p-values.**

|  | **Race  (white, AA)** | **Family income  (< or ≥$50,000)** | **Parent education (3 groups)** |
| --- | --- | --- | --- |
| **Control of Physical Activity** |  |  |  |
| Weather-related restriction of outdoor play | **0.0001** | 0.31 | **0.0001** |
| Restriction of active play indoors | **0.011** | 0.055 | 0.201 |
| Use of physical activity as a bribe | **0.0001** | **0.003** | **0.004** |
| Perceived influence on physical activity | **0.008** | 0.178 | 0.059 |
| **Control of Sedentary** |  |  |  |
| Limits on and supervision of screen media | **0.0001** | **0.0001** | **0.01** |
| Monitoring and use of TV as a threat or bribe | 0.324 | 0.049 | 0.49 |
| Monitoring and use of video games as a threat or bribe | 0.7 | 0.77 | 0.59 |
| Use of computers as a threat or bribe | **0.003** | **0.033** | 0.84 |
| Negotiation of screen media rules | 0.135 | 0.674 | 0.106 |
| Perceived influence on screen media use | **0.0004** | **0.0001** | **0.0003** |
| **Explicit Modeling** |  |  |  |
| Co-participation in physical activity | 0.088 | 0.27 | 0.37 |
| Encouragement for outside play | **0.0007** | 0.4 | 0.12 |
| Facilitation of sports and lessons | 0.76 | **0.043** | 0.62 |
| Encouragement and education to reduce screen media | 0.92 | 0.6 | 0.47 |
| Co-viewing of TV | **0.0001** | **0.013** | **0.0008** |
| Co-use of video games and computer | **0.0001** | **0.006** | **0.0001** |
| Context driven permissiveness for screen media | 0.857 | 0.855 | 0.828 |
| **Implicit Modeling** |  |  |  |
| Value of parent physical activity | 0.054 | 0.24 | 0.066 |
| Value of child sports | 0.132 | 0.89 | 0.416 |
| Value of child physical activity | **0.005** | 0.127 | 0.15 |
| Health benefits of child physical activity | 0.083 | 0.21 | 0.37 |
| Value for TV for parent | 0.14 | 0.62 | 0.035 |
| Value of child screen media | **0.0001** | **0.009** | **0.0001** |
| Entertainment and education benefits of child screen media | 0.149 | 0.114 | 0.16 |
| **Perceived Facilitators and Barriers** |  |  |  |
| Child preference for inactivity | 0.48 | 0.98 | 0.37 |
| Lack of support for physical activity from adults | 0.82 | 0.36 | 0.94 |
| Lack of self-efficacy for limiting screen media | 0.6 | 0.27 | 0.39 |
| Permissiveness for TV viewing by other adults | 0.99 | 0.54 | 0.77 |
| Permissiveness for screen media by other adults | 0.62 | 0.35 | 0.38 |
| Enforcement of screen media rules by other adults | **0.005** | **0.009** | 0.1 |
| Weather-related barriers to child physical activity | **0.008** | 0.0497 | 0.56 |
| Family consistency in beliefs around screen media | 0.052 | 0.23 | 0.47 |

P-values in red are significant (p<0.05)
